# The Effect of Calcitonin Gene-Related Peptide (CGRP) on the Cytosolic Calcium Concentration and Force in Rat Intramural Coronary Arteries

**DOI:** 10.1100/tsw.2001.443

**Published:** 2001-11-14

**Authors:** M. Sheykhzade, N.C.B. Nyborg

**Affiliations:** Department of Pharmacology, The Royal Danish School of Pharmacy, Universitetsparken 2, 2100 Copenhagen Ø, Denmark

## Abstract

The aim of this study was to investigate the mechanism of CGRP-induced relaxation in intramural rat coronary arteries. By using FURA-2 technique, cytosolic Ca-concentration ([Ca]_i_) was measured during contraction of the vascular smooth muscle with receptor-dependent agonist (tromboxane A_2_ analogue U46619) and with high concentration of extracellular potassium. At a steady state of contraction, the increase in [Ca]_i_ induced by 300 nM U46619 (100״x 14 nM, n = 7) was similar to that induced by 36 mM K (98 ״x 9 nM, n = 7). However, the active tension induced by 300 nM U46619 was significantly (p < 0.01) higher than that induced by 36 mM K. CGRP concentration-dependently (10 pM - 10 nM) reduced both the [Ca]_i_ and tension of coronary arteries precontracted with either U46619 or BAY K 8644, and also of resting coronary arteries in PSS. In 36 mM K-depolarized arteries, CGRP reduced only the tension without affecting the [Ca]_i_. In 300 nM U46619 precontracted arteries, pretreatment with 10 μM thapsigargin significantly (p < 0.05) attenuated the CGRP-induced reduction in the tension (but not [Ca]_i_). In 300 nM U46619 precontracted arteries, pretreatment with either 100 nM charybdotoxin or 100 nM iberiotoxin or 10 nM felodipine significantly (p < 0.05) attenuated the CGRP-induced reduction in both [Ca]_i_ and the tension. In contrast, 1 μM glibenclamide did not affect the CGRP-induced responses in these coronary arteries. In resting coronary arteries, only pretreatment with the combination of 1 μM glibenclamide and 100 nM charybdotoxin attenuated the CGRP-induced decrease in the [Ca]_i_ and tension, suggesting a different mechanism of action for CGRP in resting coronary arteries. We conclude that CGRP relaxes precontracted rat coronary arteries via three mechanisms: (1) a decrease in [Ca]_i_ by inhibiting the Ca influx through membrane hyperpolarization mediated partly by activation of BKCa channels, (2) a decrease in [Ca]_i_ presumably by sequestrating cytosolic Ca into thapsigargin-sensitive Ca storage sites, and (3) a decrease in the Ca -sensitivity of the contractile apparatus.

